# Barriers to male condom use in Rohingya refugee camps in Bangladesh: A qualitative study

**DOI:** 10.1016/j.lansea.2022.04.004

**Published:** 2022-05-20

**Authors:** M Mofizul Islam, Md Mashiur Rahman, Md Nuruzzaman Khan

**Affiliations:** a Department of Public Health, La Trobe University, Melbourne, Australia; b Independent Researcher, Cox's Bazar, Bangladesh; c Department of Population Sciences, Jatiya Kabi Kazi Nazrul Islam University, Bangladesh

**Keywords:** Rohingya, Contraceptive, Condoms, Family planning, Refugee, Bangladesh

## Abstract

**Background:**

Rohingya people are often called the most persecuted minority in the world. Currently, almost 800,000 Rohingya refugees live in temporary shelters in Cox's Bazar, Bangladesh. More than one-quarter of them are women and girls of reproductive age who are at increased risk of unplanned pregnancies, unsafe abortions and related complications. However, the use of contraception remains inadequate, and particularly use of condoms and male participation is scarce. This study examines the barriers to condom use as a contraceptive method among married Rohingya couples.

**Methods:**

We conducted in-depth qualitative interviews of married Rohingya men and women and thematically analysed the data.

**Findings:**

Depo Provera injections and pills are the dominant forms of contraception. Men's participation in family planning and contraceptive use is rare, and so is the use of condoms. Participants identified several barriers to condom use, including contraception being the responsibility of the women, socio-cultural issues, the stigma attached to condoms, unfamiliarity with condoms, the limitations of condoms, and issues of security in conjugal life. Health workers do not promote condoms in the same way as other contraceptive methods.

**Interpretation:**

Condom use and men's participation in contraception use are rare in Rohingya camps. The involvement of family planning workers who are males may help to promote the use of condoms and increase the method-mix options of contraceptives.

**Funding:**

La Trobe University, Melbourne, Australia.

**Research in context**

**Evidence before this study**

Only a small number of studies have examined contraception use by married couples in conflict and humanitarian settings. Data on the participation of men, particularly with regard to condom use are even scarcer. While some previous studies found women mostly use contraception, little is known about the barriers to condom use by married men in the context of the displaced Rohingya people.

**Added value of this study**

This study provides an understanding of the barriers to male condom use among married Rohingya couples in the refugee camps in Bangladesh. Some of the barriers, such as contraceptive use being a responsibility of the women and issues of security in conjugal life are unique to this population. The involvement of male Rohingya family planning workers for outreach services can promote men's participation in contraception and condom use.

**Implications of all evidence available**

The limited use of contraceptives and family planning services among displaced people is a critical public health issue, particularly at a time when forcibly displaced populations grew substantially throughout the world. Given that the practice of contraceptive use and access to family planning services in refugee settings are context-specific, the identified barriers should be taken into consideration for designing appropriate programs in the Rohingya camps in Bangladesh and elsewhere.

Alt-text: Unlabelled box

## Introduction

Abound 800,000 Rohingya people are living in refugee camps in Cox's Bazar, Bangladesh, after having been displaced from Rakhine state, in western Myanmar. These people left their motherland, Myanmar, following violent military operations. Although the exodus began in 1970, most of the Rohingya people arrived in 2017, after the most recent round of operations by Myanmar military, which the International Court of Justice later declared to be genocide. To save their lives, the Rohingya people fled to other countries in South and Southeast Asia, including Bangladesh, India, Malaysia, Thailand and the Philippines. As Rakhine State is at the border of Bangladesh, it received the highest numbers.^1^ While living in Myanmar, these people had been deprived of their citizenship and the fundamental rights of freedom of movement, education and health care.^2^^,^^3^ Their community had been deliberately left behind, based on the allegation that they were the descendants of Bengali people who had illegally migrated from Bangladesh many years earlier. This large group of Rohingya refugees now lives in temporary and crowded shelters, which are located in a hilly area in Cox's Bazar, Bangladesh. The living conditions in the camps are inadequate for meeting their needs and also unhealthy. The average number of people per household is 4.5 and they live mainly in small makeshift shelters of 14 square meters in size that were built out of bamboo and tarpaulins. Because Bangladesh is not a signatory to the 1951 Refugee Convention, the Rohingya people are treated as displaced citizens from Myanmar. They are not officially allowed to work in Bangladesh or to live outside the camps. They are entirely dependent on food and other essential supplies that are provided by national and international donor organizations such as World Food Programme and United Nations Population Fund.^4^

The need for family planning among displaced people is an issue that has been well documented.^5^^,^^6^ A sudden displacement often results in loss of livelihoods, lack of education and heightened insecurity for unmarried girls, and this may contribute to increases in child marriages.^7^^,^^8^ In the Rohingya camps, more than one-quarter of refugees are women and girls of reproductive age.^9^ Also, the overall health of the refugees in such camps is generally poor.^10^, ^11^, ^12^, ^13^, ^14^, ^15^, ^16^ Nutritional deficiencies are highly prevalent among Rohingya refugees, especially among children. Nearly half of the children aged 6-59 months suffer from stunting and anaemia, and about one-fourth had Global Acute Malnutrition.^17^ Around 10% of Rohingya women were undernourished.^18^ A need assessment survey conducted in 2018 found that 51.5% of Rohingya people had hypertension and 14.2% had diabetes.^17^ More than a third (36%) of Rohingya refugees were suffering from posttraumatic stress disorder. Symptoms of depression (>80%) and suicidal thoughts (13%) were also dominant in the Rohingya community.^19^ It is anticipated that tuberculosis is also prevalent because of poor living conditions and overcrowding and the high prevalence of tuberculosis in Myanmar.^20^ Although the government of Bangladesh, together with several national and international organisation, offer family planning and contraception services, these are inadequate. These situations increase the risk of unplanned pregnancies, unsafe abortions and related complications,^21^ which add further hardships to the already dreadful situations. Besides, since they live in congested conditions, having a large family is obstructive to even a minimum standard of life, which highlights the importance of using family planning services and contraception.

While they were in Myanmar, many of these people did not use family planning clinics, fearing that the authorities would give them harmful medications to hamper reproduction and hinder population growth.^22^ Their relatively low levels of formal education and their religious and conservative beliefs create further barriers to contraceptive use. Despite current efforts to promote the use of contraception and family planning services, their overall use remains relatively low.^23^ For instance, a recent paper reported that about half of the women of reproductive age were not using contraception.^24^ Some other studies have also found that it is generally the women who use contraception, predominantly injections and oral pills, and that there is a very low prevalence of condom use among males.^25^^,^^26^ The use of condoms can reduce the pressure from women and also increase the method-mix. A broad method-mix suggests that the population has access to a range of different contraceptive methods. Besides, condoms are relatively cheap, can be accessed without a prescription, and only contraceptive method that prevents both pregnancy and sexually transmissible infections. They are also safe to use while a woman is breastfeeding. Therefore, it is critical to understand the reasons for this low participation in contraceptive use by men, in particular with regard to condoms. However, to our knowledge, no studies have yet examined these issues. Therefore, this study examines why the participation of men in contraceptive use and particularly condom use is low in the Rohingya camps.

## Methods

We administered face-to-face qualitative interviews. The potential participants were identified by three local Rohingya volunteers who live in that settlement. One of them is a Rohingya woman who interviewed the female participants. She was assisted by an experienced local Bangladeshi woman who speaks both Rohingya and Bengali dialects. A total of 14 women and 10 men were interviewed by using an open-ended questionnaire. A convenience sampling approach was used for selecting participants. Men and women of reproductive age who were living with their spouses at the time of the survey were targeted for the interview. To ensure privacy, the interviewers endeavoured to organise one-on-one interviews in participants’ houses and without the presence of others. As result, some interviews needed several visits and attempts by the interviewers. The questionnaire covered a range of items including participants’ past and current history of contraception use, reasons for preference to the methods they used and had specific questions about condom use as a contraception method. Data saturation was ensured with the last few interviews, which provided no new information. The questionnaire was piloted among two participants to ensure its appropriateness. All participants were also asked the reasons for contraception use. About 30 ∼ 45 minutes were needed to run an interview. The second author (MMR) supervised the data collection.

The participants were informed of the purpose of the study and about their voluntary participation. Participants were informed that they would remain anonymous and would be assigned pseudonyms in any transcripts of the interview data and therefore they would not be identifiable. All participants had provided informed consent prior to the interviews. Ethical Approval for the survey was given by the Institute of Biological Science, University of Rajshahi, Bangladesh (approval: 123/320/IAMEBBC/IBSc). The study also received approval from the Refugee Relief and Repatriation Commissioner's Office in Cox's Bazar, Bangladesh.

### Data analysis

All interviews were audio-recorded and transcribed into Bengali. Transcripts were reviewed line-by-line and checked for accuracy and completeness. The first author read the content several times while observing general patterns in participants’ responses. We started with an open coding approach and then revised the codebook continuously as we coded additional transcripts. We manually coded the recurrent ideas and sorted them into categories and themes. Themes were developed inductively, keeping the focus of the analysis on the explicit meanings of the information provided by the participants. Then we focussed on connecting themes and linked the core themes to the aims of the study through a process of collaborative analysis. We manually organised and analysing data. Only those parts of Bengali transcriptions needed for quotes were translated to English. Hard copies of the interview transcripts were stored securely in a locked cabinet.

## Results

The demographic variables of the participants are shown in Table 1. The median age of the participants was 25.5 years with an age range of 19-45 years. All participants had given birth to at least one child. At the time of the survey, 62.5% of participants were using contraception and only two men (8.3%) reported that they were using condoms. Although the vast majority (75%) have heard about condoms as a contraceptive method, only 20.8% ever used them.

**Table 1:** Demographic characteristics of the participants.

Variable	Number / percentage
Number of participants	24
Sex	
Male	14
Female	10
Median age	25.5
Age range	19 – 45
Average number of children	2.5
Currently using any contraception	62.5%
Ever heard about condoms	75%
Ever used condoms as contraception	20.8%

**Figure 1:**
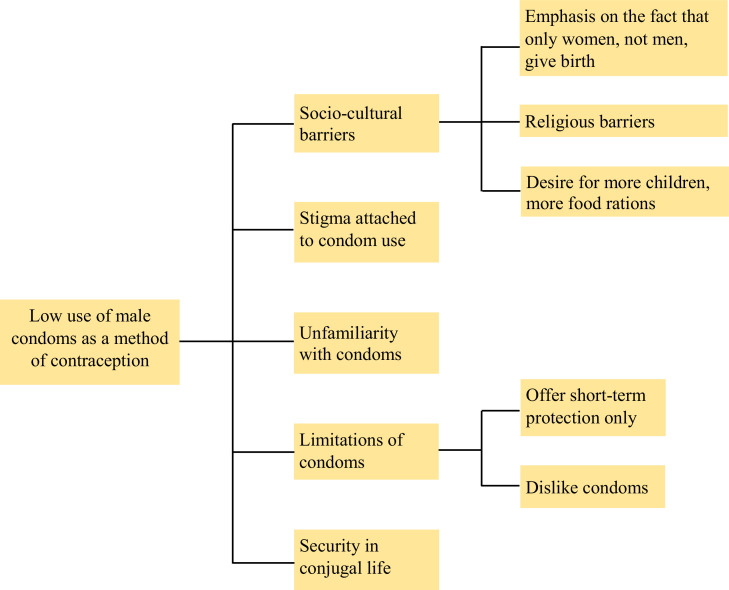
Barriers to the use of condom by males as a method of contraception in Rohingya camps, Cox's Bazar, Bangladesh.

This study aimed to identify the barriers to men's participation in contraceptive use and relatively low use of condoms as a contraceptive method in the Rohingya camps, Cox's Bazar. We found five themes, namely, (i) socio-cultural barriers, (ii) the stigma attached to condoms, (iii) unfamiliarity with condoms, (v) the limitations of condoms and (vi) issues of security in conjugal life. The emphasis on the fact that women, not men, give birth, religious barriers and desire for more children and more food rations are the subthemes for socio-cultural barriers. The ability to offer only short-term protection and disliking of condoms are two subthemes of the limitations of condoms (Figure 1). A detailed description of themes and subthemes and associated quotes are presented below:

### Socio-cultural barriers

#### The emphasis on the fact that it is the women, not the men, who give birth

Social perceptions and norms were found to be barriers to men's participation in the use of contraception, particularly condoms. Both men and women highlighted the gender norms and social expectations that assign the roles of childbirth and child-rearing to women. Birth spacing and contraceptive use were also considered to be within this domain, and involving men in family planning services was perceived by some as unusual. Only a small subset of men stated that they believed family planning to be a shared responsibility and almost all the women believed this to be a common perception among men and the broader society, as indicated by this quote from a participant:

“*I use pills and haven't tried anything else as I think pills suit my body well. If pills didn't suit my body, and I asked my husband to use condoms, I believe that he would do so. However, most men in the camps believe that since it is women, not men, who give birth to babies, women should be the ones to use contraception*”.

Due to this social expectation and common practice, most women never ask their husbands to use condoms, instead, they start using contraception because unless they maintain reasonable birth spacing, they will get sick. Due to this proactive role by women in using contraception, men do not need to use condoms or any other form of contraception. One participant stated the following:

*“I never asked or wanted to ask my husband to use contraception and relieve me from using pills. It's normal in our community that contraception is for women. But, if men started using contraception, it would surely help a lot of women and reduce the substantial health burden we face”.*

#### Religious barriers

Religious directives are a barrier to all types of contraception, including condoms, since hindering the natural course of childbirth is believed to contradict such directives (Figure 1). Even among those who use contraception, there remains a hesitation about the moral legitimacy of its use. Here is what one husband mentioned about his wife's contraceptive use.

*“The religion, Islam, doesn't support the use of contraception. I am a practicing Muslim, and there is a hesitation in my mind about contraceptive use. After giving birth to three babies, my wife became very skinny and physically weak. Recently, a doctor advised me to use condoms for a few months. I may eventually use condoms, but I can't free my mind from this hesitation”.*

One woman who recently started using Depo-Provera (Depo) but kept it a secret from her husband for a while stated the following:

“*My husband is a religious person. I don't think he will ever use condoms or anything else. He doesn't like me using contraception. Currently, I use Depo, but I kept it a secret from him for quite a while. He was invited to attend family planning-related meetings that are organised for men, but he never went to any. I asked him once the reasons why he avoided these meetings, and he replied that he doesn't believe in contraception and asked me not to insist that he join the meetings*”.

Although participants believe, as per religious directives, that all contraceptives are prohibited, there appear to be particular barriers that are associated with condoms than with other forms of contraception such as Depo or pills. One woman explained this as follows:

“*I use Depo. But I think Muslims should not use condoms. They are for the people of other religions*. Condoms don't allow my husband to get close to me to the extent that he desires. Condoms don't allow me to fulfil my responsibility as a wife”.

However, the men who were supportive of contraceptive use did not mention the religious barriers. Some of these men cited the current congested living conditions, uncertain futures, and the benefits of keeping family sizes relatively small. One man stated:

*“Life in the camp is difficult. Most houses are too small to accommodate a relatively large family. I am happy with my two children. If ever the situation changes, we may think about another baby, but not now”.*

#### Desire for more children and food rations

Some families hold traditional views and want multiple children as part of their roles in maintaining their inheritance. It was traditional to have many children in a family, but this has changed substantially in recent times. Also, the amount of food and other groceries that a household receives is determined by the number of individuals (Figure 1). Therefore, having multiple children increases the amount of food that is supplied, and this could be a reason why some families do not use contraception. Since men are generally considered to be the heads of the families in the Rohingya community, if they hold the traditional views about having large families and want bigger rations, they are unlikely to use condoms. They may not want their wives to use contraception, and even if they allow them to do so this is likely to be for birth spacing. Here is how two female participants explained these factors:

*“My husband doesn't like using contraception and thinks we need more children. I don't know whether part of his desire for more children is related to the rations we receive. The more children a household has, the more rations it receives. So, having several children may be helpful. Also, my husband is a practicing Muslim. He doesn't believe in contraception. But I started using pills, as I don't want too many babies within short intervals”.*

*“In the past, having multiple children in a family was common. But nowadays, people's thinking has been changed. My husband holds traditional views, and he thinks we need children to conserve our inheritance. He doesn't like me using contraception, although he never put any pressure on me once I started using Depo.”*

### Stigma attached to condom use

A substantial amount of stigma is attached to condom use. Some women believe that people who use condoms during sexual intercourse do so because they are in illegitimate sexual relationships (Figure 1). Here are two quotes from women in response to a question regarding whether their husbands ever use condoms:

“*I have heard that evil women want their sexual partners to use condoms to prevent pregnancies from extramarital sexual intercourse. We are not like them, so why should we use condoms?”*

“*Even if my husband wants to use condoms, I wouldn't allow him to do so. Whenever I think about condoms, I feel awful. I would prefer to use other contraceptives if we needed to use them, rather than allow my husband to use condoms*”.

Condoms have acquired a bad name, and some people also associate them with promiscuity, probably because they are advertised extensively in educational programmes in the media for the prevention of the Human Immunodeficiency Virus and other sexually transmitted infections. In fact, some Rohingya men and women consider condoms to be a symbol of extramarital sexual relationships. Indeed, some men find collecting condoms from clinics or purchasing them from shops embarrassing. The following quote from one man illustrates his feelings about collecting/purchasing condoms:

*“I would feel shy about asking for condoms in health clinics or purchasing them from shops. However, Depo or pills are considered normal, and I feel that women should not feel shy about using them. I have never used condoms; all I know is that men don't like using condoms as they take away the sexual pleasure”.*

### Unfamiliarity with condoms

Many women said they were unfamiliar with condoms and about how to use them (Figure 1). Some women had never seen a condom. Depo and pills are the most prevalent methods. Condoms are not promoted in the same way as other contraceptive methods. However, all men were aware of condoms, even though only a few had ever used them. Here are several quotes from some married Rohingya men and women that indicate their familiarity or lack of familiarity with condom use:

*“I never heard about condoms from anybody. People often discuss Depo, pills and IUDs [intrauterine devices], but they do not talk about condoms. I have never even seen a condom, so I have no idea what they look like. I have used pills but have never thought about anything else. Men don't use contraception. I had never thought about my husband using condoms”.*

*“When I visit health clinics, I receive information about Depo, pills and IUD implants, but nobody ever told me anything about condoms for my husband. Currently, I am not using anything as we are trying to have our second baby”.*

When the data collector explained how condoms are used, this woman made the following comment:

*“I can't imagine my husband using condoms. This sounds terrible. I learned about pills and Depo from my mother, and these are the methods that I know that women use. We have heard that pills and condoms both reduce fertility, but I don't know any couples around me who use condoms”.*

### Condom-related barriers

#### Do not offer long-term protection

Most men and some women identified condoms as short-term contraception. They compared condoms to Depo or intrauterine devices and stated that there has to be a constant supply of condoms. They identified the need to have such a supply of condoms as a barrier to their use. Here are two quotes from a man and a woman, respectively:

*“Depo gives protection for a long time; for instance, for three months. Therefore, while Depo can offer longer-term protection, a condom is something you need to use every time you have sexual intercourse. If Depo suits a woman, then it is quite likely that her husband won't use condoms”.*

*“A nurse had told me that she had noticed some bugs in a clinical sample taken from my body, and she advised me to ask my husband to use condoms so that he wouldn't get the same bugs. I was given around 30 condoms. My husband used them all up about a month ago, and he is not using anything now, as I haven't had time to go to the clinic to collect another lot”.*

When asked whether they had sexual intercourse without using condoms after the condom supply had run out, she responded: “*Yes, we did; my husband didn't care about the bugs*.”

When asked if her husband could go to hospitals to collect condoms, she replied: “He won't go to hospitals or shops to collect or purchase condoms. He would find this embarrassing. He would continue using nothing until or unless I go and get some for him.”

#### Dislike of condoms

Participants identified disliking condoms as a barrier, citing a number of reasons. These included the flavour of condoms, difficulty in finding the appropriate sizes, a loss of sensation and sexual pleasure, difficulty maintaining an erection and issues with the storage and disposal of the condoms. Here is a quote that illustrates how one woman felt:

*“I know how a condom is used; men use them. I use pills because my husband did not like the smell of condoms.”*

However, even though some participants said they disliked condoms, they mentioned that they still used them, mainly because there were no other suitable contraceptive options for men. One man stated the following:

*“Both my wife and I dislike condoms. But we use both pills and condoms. Using pills for a long period of time is harmful to my wife's health. So, we kind-of use these two methods sequentially. For example, I use condoms for a couple of months, and then my wife starts using pills for a few months. Unfortunately, there is no other suitable contraception for men”.*

Traditionally, although the use of contraception by newly married Rohingya couples is rare before they give birth to their first babies, the new generation of couples is more likely to be aware of the benefits of contraceptive use and the advantages and limitations of the different methods. They are also likely to switch to methods based on their circumstances. Here is a quote from a newly married man:

*“Currently, I use condoms. I have a friend who works in a pharmacy. He advised me to use condoms right from the beginning of my marriage. I used them for seven days initially, and then my wife started taking pills. However, after a certain period of time, my wife started getting health problems from using contraceptive pills. We discussed this with a doctor, and I started using condoms again as per his advice”.*

### Security in conjugal life

Female participants preferred using contraception themselves than asking their husbands to use condoms. This observation is consistent even among those women who were having health problems from using pills or Depo. Some women tried to hide the health issues that developed from their contraceptive use, fearing that their husbands might not like this and might even consider marrying again [a second wife], as is illustrated in the two quotes below:

*“My husband does not like to attend family planning meetings or to use condoms. So, I use pills, although I get headaches and feel tired. I have been planning to switch to Depo if these health problems persist. If I keep telling him about these physical weaknesses, I fear he may consider making another marriage, although I don't know whether he really would.”*

Sometimes women do not use contraception and do not want their husbands to use it either; this occurs particularly before they have given birth to their first babies, because they believe that children make conjugal relationships stronger and enhance their husbands’ commitment (Figure 1). Here is a quote from a female participant that illustrates this point:

*“It's common for couples not to use contraception immediately after marriage because they want children. We lost our first baby, who died shortly after his birth. Why should my husband or I use protection now? I may use birth control methods after the birth of my next baby and before I get pregnant again. Having children brings security in conjugal life and my husband will take care of me more once we have babies”.*

## Discussion

The findings of this study provide an overview of the barriers to the use of condoms by Rohingya men living in the refugee camps in Cox's Bazar, Bangladesh. Among married couples, the use of condoms by males is less prevalent than the use of Depo and pills by women. Married men and women identified several barriers to condom use, such as contraception being the responsibility of the women, socio-cultural issues, the stigma attached to condoms, unfamiliarity with condoms, the limitations of condoms and issues of security in conjugal life (Figure 1). Also, women's limited autonomy and decision-making power impact their ability to negotiate contraception use in general and the use of condoms in particular. Although we are unable to state precisely whether reducing these barriers will help the reproductive health and family planning of individual couples, removing misunderstanding and making the couples aware of both the benefits and the limitations of condoms may help to increase the choices they have regarding contraceptive methods and reduce the excessive pressure that is placed on women.

Some men are likely to be responsible for the barriers, which contributes to low participation in contraceptive use among these males. Our results suggest that in most cases women start contraceptive use because they want to control the number of children they have and their birth spacing, and some even keep their contraceptive use secret from their husbands. However, the active involvement of men with regard to contraceptive use is crucial for ensuring two interrelated goals: encouraging the use of male methods of contraception and expanding the involvement of men in making decisions around family planning. The awareness of men regarding the benefits of contraceptive use may change the circumstances of the married couples. Spousal communication can be an important strategy in increasing men's participation in reproductive health, and communication may enable husbands and wives to assess their circumstances and differing attitudes toward contraceptive use.

A key observation made through this study is that the use of condoms is not promoted as much as that of Depo or pills (Figure 1). Although further investigation is needed to identify the reasons for this, one possibility is that health workers fear that condoms may not be used properly or indeed at all. Local media reports suggest that when the Rohingya refugee women were initially given oral pills, they would take them home but then throw them away.^25^ It is possible that this fear influences the preference among health workers for Depo, which offers birth control for longer durations of time. The fact that there is relatively little awareness about the use of condoms and the perception that only women should use contraception may be other reasons. Also, the somewhat high failure rate, around 13%, that occurs with regular use of condoms,^27^ may discourage health workers from recommending them, and further research is needed on this.

Family planning and contraceptive use are matters of individual choice. The authors do not intend to promote the use of condoms among males over any other forms of contraception. However, increasing the method-mix of contraception is useful and results in a range of different contraceptive methods and, thereby, better services offered.^28^ Several female participants mentioned the health problems they had as a result of using pills and Devo. Some of them had even had to put their contraceptive use on hold for varied periods of time, while many continued using them in spite of constant health problems. The use of condoms increases the method-mix options for couples. Many of the barriers to the use of condoms among men can be overcome by involving family planning workers who are males. Based on the information we received from the field, almost all family planning workers are currently women.

Our findings suggest that some people associate condoms with promiscuity or extramarital sexual intercourse and there was a substantial degree of stigma attached to condoms, particularly among the female participants (Figure 1). The use of condoms for protection against sexually transmitted infections has also added further stigma, creating additional barriers to the access to and usage of condoms. Previous studies suggest that because of the proactive role of health and family planning workers in the camps there has been considerable improvement in the acceptability and use of contraception among Rohingya people. Indeed, Bangladesh's success in family planning programs is well recognised.^29^ Therefore, we believe it is possible for health workers to reduce the stigma that is attached to condoms. However, the process of obtaining condoms should be improved and made to cause less embarrassment to those who want them.

As our results suggest, religious prohibition is a key barrier to the use of contraception in the Rohingya community, in general, and condoms, in particular. This observation is consistent with the literature.^30^ However, recent literature suggests that this barrier has been removed substantially using another religious directive that all children deserve a decent life, which entails that their number may need to be limited and therefore the use of contraception is acceptable. Other barriers, such as the social expectations that assign the roles of childbirth and child-rearing to women and the responsibilities of contraception use to them, need to be reduced.. Also, it is concerning that some people avoid using condoms despite health workers’ recommendations in the context of diagnosed sexually transmitted infections. Women's desire to fulfill the wifely responsibility of offering sexual pleasure to their husbands and hence avoiding condoms also warrant attention.

This study has some limitations. Contraceptive use is a sensitive topic, and some participants may not have disclosed everything clearly. Also, we used multiple languages from questionnaire design to final report writing. The questionnaire was developed in the Bengali language. But the interviews were conducted in the Rohingya language and recordings were transcribed and analysed in Bengali and finally the findings were translated into English to write this paper. However, to minimise this drawback we involved experienced data collectors and transcribers. It is possible that this perception is held by a small subgroup of Rohingya people and so our sample missed them. The use of three languages may have contributed to missing or loss of meanings in questions, responses and interpretations of findings. Lastly, although the interviewers endeavoured to ensure the privacy of the respondents by having one-on-one interviews either in participants’ houses or outside, without the presence of others, it has not been always possible particularly when the female participants had small children.

## Conclusion

Promoting positive changes in the attitudes toward condom use among those living in the Rohingya camp requires an understanding of the perceptions of the people, their current practices and social and religious factors. This paper offers some understanding of the barriers to the use of condoms by married Rohingya couples. Several barriers have been identified; namely, contraception being the responsibility of the women, socio-cultural issues, the stigma attached to condoms, unfamiliarity with condoms, the limitations of condoms and issues of security in conjugal life. Some of these barriers, including social stigma and misunderstandings attached to the use of condoms, can be addressed with reasonable effort. The involvement of men in reproductive services and having family planning workers who are males may also help to promote the use of condoms and increase the method-mix options of contraceptives.

## Contributors

MMI designed the study and analysed the data and wrote the first draft of the article. MMR supervised the data collection. MMR and MNK verified the underlying data. All authors edited and approved the final version of the Article.

## Data sharing statement

De-identified data that support the findings of this study are available from the corresponding authors upon reasonable request following the publication of this article. Approval may be required from the Office of the Refugee Relief & Repatriation Commissioner, Cox's Bazar, Bangladesh.

## Authors’ information

The lead author (MMI) is a senior lecturer at the department of public health at La Trobe University, Melbourne. MMI has an MSc in public health from the University of Sydney and a Ph.D. in public health and community medicine from the University of New South Wales, Australia. He received the necessary education and training for conducting qualitative research. The second author (MMR) completed a master's degree in social science. MMR has working experience in the population health of the Rohingya community in Cox's Bazar, Bangladesh. The last author (MNK) is an assistant professor at the Department of Population Sciences, Jatiya Kabi Kazi Nazrul Islam University, Bangladesh. He completed his Ph.D. in the discipline of clinical epidemiology and medical statistics from the University of Newcastle, Australia.

## Declaration of interests

All other authors declare no competing interests.

## Appendix Supplementary materials

Image, application 1

Image, application 2
