# Supplementary material for: Barriers to male condom use in Rohingya refugee camps in Bangladesh: A qualitative study
Source: Lancet Reg Health Southeast Asia. 2022 May 20;2:100008. doi: 10.1016/j.lansea.2022.04.004 (PMC10305886; doi:10.1016/j.lansea.2022.04.004)
Supplement: Supplementary file 1 [file mmc1.docx]

*Disclaimer: This translation in [language] was submitted by the authors and we reproduce it as supplied. It has not been peer reviewed. Our editorial processes have only been applied to the original abstract in English, which should serve as reference for this manuscript.*

**Abstract in Bengali**

রোহিঙ্গা জনগোষ্ঠী বিশ্বের সবচেয়ে নির্যাতিত সংখ্যালঘু জাতি হিসেবে পরিচিত । বর্তমানে প্রায় আট লক্ষ রোহিঙ্গা শরণার্থী বাংলাদেশের কক্সবাজারে অবস্থিত অস্থায়ী আশ্রয়শিবিরে বসবাস করছে । তাদের মধ্যে এক-চতুর্থাংশের বেশি প্রজনন বয়সের মহিলা এবং কিশোরী, যারা অপরিকল্পিত গর্ভধারণ, অনিরাপদ গর্ভপাত এবং এ সম্পর্কিত জটিলতার ঝুঁকিতে রয়েছে, যা পরিবার পরিকল্পনা পরিসেবা এবং গর্ভনিরোধক ব্যবহারের গুরুত্ব তুলে ধরে । পূর্ববর্তী গবেষণায় গর্ভনিরোধক পদ্ধতি হিসাবে কনডমের ব্যবহার খুব কম পাওয়া গেছে এবং শুধুমাত্র মহিলারা পরিবার পরিকল্পনা সেবা ব্যবহার করে বলে জানা গেছে । বিবাহিত মহিলা ও পুরুষদের কাছ থেকে গুণগত তথ্য সংগ্রহ করেছি। থিম্যাটিক বিশ্লেষণের মাধ্যমে গুণগত তথ্যসমূহ হতে মুল বিষয়বস্তু উদঘাটন করা হয়েছে। আশ্রয়শিবিরের মহিলাদের মধ্যে দেবপ্রভেরা (ডেপো) ইনজেকশন এবং খাবার বড়ি গর্ভনিরোধের বহুল ব্যবহৃত পদ্ধতি । পরিবার পরিকল্পনা এবং গর্ভনিরোধক ব্যবহারে পুরুষদের অংশগ্রহণ বিরল । গবেষণায় অংশগ্রহণকারীরা কনডম ব্যবহারে বেশকিছু প্রতিবন্ধকতা চিহ্নিত করেছেন যার মধ্যে রয়েছে গর্ভনিরোধক মূলত মহিলাদের দায়িত্ব হিসেবে বিবেচনা করা, সামাজিক-সাংস্কৃতিক অবস্থা, কনডম সম্পর্কিত কুসংস্কার, কনডমের অপরিচিতি, পদ্ধতি হিসেবে কনডমের সীমাবদ্ধতা এবং দাম্পত্য জীবনের নিরাপত্তা । স্বাস্থ্যকর্মীরা অন্যান্য গর্ভনিরোধক পদ্ধতির তুলনায় কনডমের প্রচার ও ব্যবহারের সুপারিশ অনেক কম করেন । পুরুষ পরিবার পরিকল্পনা কর্মী নিয়োগের মাধ্যমে এবং কনডম ব্যবহারকে উৎসাহিত করে গর্ভনিরোধক পদ্ধতির-মিশ্রণ ও প্রকার বাড়িয়ে তোলা সম্ভব ।
