# Supplementary material for: Barriers to male condom use in Rohingya refugee camps in Bangladesh: A qualitative study
Source: Lancet Reg Health Southeast Asia. 2022 May 20;2:100008. doi: 10.1016/j.lansea.2022.04.004 (PMC10305886; doi:10.1016/j.lansea.2022.04.004)
Supplement: Supplementary file 2 [file mmc2.pdf]

ইন্টারভিউ গাইড

নব্য বিবাহিত যুগলদের প্রজনন স্বাস্থ্য সম্পর্কিত প্রশ্নের গাইড (সেবা প্রদানকারী জন্য)

ভূমিকাঃ

আস্‌সালামু আলাইকুম/আদাব,

আমার নাম: --। আমি একটি বিশ্ববিদ্যালয়-ভিত্তিক গবেষণার তথ্য সংগ্রহের জন্য এসেছি। সংগৃহীত তথ্য নব্য বিবাহিত মহিলা ও পুরুষদের স্বাস্থ্য ও পরিবার পরিকল্পনা সেবা সম্পর্কে ধারনা পেতে সহায়তা করবে। জরিপ কাজে আনুমানিক ২০-৩০ মিনিট সময় লাগবে। এই জরিপে অংশগ্রহণ সম্পূর্ণরূপে আপনার ইচ্ছার উপর নির্ভরশীল। আশা করবো আপনি এই জরিপে অংশগ্রহণ করবেন, কারণ আপনার মতামত এই জরিপের জন্য অত্যন্ত গুরুত্বপূর্ণ। আমি যদি আপনাকে কোনো প্রশ্ন জিজ্ঞাসা করি যা আপনি উত্তর দিতে চান না, তাহলে আমাকে বলবেন, সেক্ষেত্রে আমি অন্য প্রশ্নে চলে যাব। তা ছাড়াও যে কোনো সময় আপনি সাক্ষাৎকার প্রদান বন্ধ করে দিতে পারেন।

কেন জরিপটি করা হচ্ছে?
এই জরিপের উদ্দেশ্য হচ্ছে নব্য বিবাহিত মহিলা ও পুরুষদের জন্মনিয়ন্ত্রণ পদ্ধতি সম্পর্কিত প্রয়োজনীয় তথ্য/উপাত্ত সংগ্রহ করা।

অংশগ্রহণে সম্মত হলে আপনাকে কী করতে হবে?
নির্ধারিত কিছু বিষয়ের উপর আপনার মূল্যবান মতামত প্রদান করতে হবে। আপনি যদি কিছু প্রশ্নের উত্তর দিতে অস্বস্থি বোধ করেন তাহলে ওই সব প্রশ্নের উত্তর প্রত্যাখ্যান করতে পারেন।

এই গবেষণায় অংশগ্রহণের ঝুঁকি কী?
এই জরিপে অংশগ্রহণ করে আপনি কোনো ঝুঁকির সম্মুখীন হবেন না।

গোপনীয়তাঃ
আপনার প্রদত্ত তথ্য সম্পূর্ণভাবে গোপন রাখা হবে। এটা শুধুমাত্র গবেষণার কাজেই ব্যবহৃত হবে এবং গবেষকগণই কেবল আপনার উত্তরসমূহ জানতে পারবেন।

এই গবেষণায় অংশ নেওয়ার জন্য কি কোনো আর্থিক সুবিধা প্রদান করা হবে?
এই জরিপে অংশগ্রহণ সম্পূর্ণরূপে আপনার ইচ্ছার উপর নির্ভরশীল এবং অংশগ্রহণের জন্য আপনাকে কোনো আর্থিক সুবিধা প্রদান করা হবে না।

অংশগ্রহণে অসম্মতি বা প্রত্যাহারের অধিকারঃ
এই জরিপে অংশগ্রহণ সম্পূর্ণরূপে আপনার ইচ্ছার উপর নির্ভরশীল। আপনি চাইলে কোনো বিশেষ একটি প্রশ্নের উত্তর নাও দিতে পারেন।

টার্গেট গ্রুপঃ পুরুষ ও মহিলা যারা গত তিন বছরের মধ্যে জন্মনিয়ন্ত্রণ পদ্ধতি ব্যবহার করেছেন।

প্রশ্নঃ আপনি অথবা আপনার স্বামী/স্ত্রী কি গত তিন বছরের মধ্যে কোনপ্রকার জন্মনিয়ন্ত্রণ পদ্ধতি ব্যবহার করেছেন অথবা এখন করছেন?

(যদি উত্তর হয়ে থাকে না, অথবা যদি উত্তর হয়ে থাকে কোন প্রাকৃতিক পদ্ধতি যেমন, যৌনকাজ হতে বিরত থাকা, উত্তোলন/অপসারণ (withdrawal), তবে ঐ ব্যক্তি এই ইন্টারভিউ-এর জন্য উপযুক্ত নয়।)

প্রশ্নঃ বর্তমানে আপনার বয়স কত? (আনুমানিক)

প্রশ্নঃ আপনি কত বছর আগে বিয়ে করেছিলেন?

প্রশ্নঃ আপনাদের ছেলেমেয়ে কয়জন? সবচেয়ে ছোট সন্তানের বয়স কত?

প্রশ্নঃ (মহিলাদের জন্য) আপনি কি এখন গর্ভবতী?

প্রশ্নঃ বর্তমানে অথবা গত তিন বছরের মধ্যে আপনারা কী কী জন্মনিয়ন্ত্রণ পদ্ধতি ব্যবহার করেছিলেন বা করছেন?

প্রশ্নঃ গত তিন বছরের মধ্যে জন্মনিয়ন্ত্রণ পদ্ধতি হিসাবে আপনারা (অর্থাৎ আপনি অথবা আপনার স্বামী/স্ত্রী) কি কনডম ব্যবহার করেছিলেন?

প্রশ্নঃ আপনারা কেন কনডম ব্যবহার করেননি? দয়াকরে কারণগুলো পরিষ্কার করে বলেন।

প্রশ্নঃ জন্মনিয়ন্ত্রণ পদ্ধতি হিসাবে ক্যাম্পে কনডম ব্যবহারের প্রচলন কীরকম? আপনার ধারনা থাকলে বলুন।

প্রশ্নঃ ক্যাম্পে কনডম কি সরবরাহ করা হয়? অর্থাৎ আপনারা যদি কনডম ব্যবহার করতে চান তাহলে কি কনডম সংগ্রহ করতে পারবেন?

প্রশ্নঃ কীভাবে পারবেন, কোথা থেকে সংগ্রহ করবেন?

প্রশ্ন **(পুরুষদের জন্য)**: আপনি যদি কনডম ব্যবহার করতে চান, তবে কি আপনার স্ত্রী তাতে আপত্তি করবে বলে মনে করেন?

প্রশ্ন **(মহিলাদের জন্য)**: আপনি যদি কনডম ব্যবহার করতে বলেন, তবে কি আপনার স্বামী তাতে আপত্তি করবে বলে মনে করেন?

প্রশ্নঃ যদি আপত্তি করবে বলে আপনি মনে করেন, তবে তার কারণ কী? দয়াকরে কারণগুলো বলুন।

প্রশ্নঃ আপনি কি মনে করেন বার্মার তুলনায় ক্যাম্পে জন্মনিয়ন্ত্রণ পদ্ধতি ব্যবহারের পরিমাণ বেড়েছে?

প্রশ্নঃ (যদি উত্তর হ্যাঁ অথবা না হয়ে থাকে) এর কারণগুলো কী কী বলে আপনি মনে করেন?

প্রশ্নঃ পরিবার-পরিকল্পনা পদ্ধতি হিসাবে আপনাদের কি কখনো কনডম ব্যবহার করার পরামর্শ দেয়া হয়েছিল?

প্রশ্নঃ (যদি উত্তর হ্যাঁ হয়ে থাকে) কে এই পরামর্শ দিয়েছিল? আপনারা কি পরামর্শ মোতাবেক কনডম ব্যবহার করেছিলেন? ব্যবহার করে থাকলে কতদিন ব্যবহার করেছিলেন? আর যদি না করে থাকেন তার কারণ কী?

প্রশ্নঃ ক্যাম্পে জন্মনিয়ন্ত্রণ পদ্ধতি মূলত: মহিলারাই ব্যবহার করে থাকে? এর কারণগুলো কী কী বলে আপনি মনে করেন?

প্রশ্নঃ আপনি কি মনে করেন পুরুষরাও জন্মনিয়ন্ত্রণ পদ্ধতি ব্যবহার করা উচিৎ? কেন এমনটা মনে করেন?

আপনার মূল্যবান সময় ও ইন্টারভিউতে অংশগ্রহণের জন্য আপনাকে অসংখ্য ধন্যবাদ।

----ঃ----
